# A High Excision Potential of TALENs for Integrated DNA of HIV-Based Lentiviral Vector

**DOI:** 10.1371/journal.pone.0120047

**Published:** 2015-03-17

**Authors:** Hirotaka Ebina, Yuka Kanemura, Naoko Misawa, Tetsushi Sakuma, Tomoko Kobayashi, Takashi Yamamoto, Yoshio Koyanagi

**Affiliations:** 1 Laboratory of Viral Pathogenesis, Institute for Virus Research, Kyoto University, 53 Shogoin-kawara-cho, Sakyo-ku, Kyoto 606-8507, Japan; 2 Department of Mathematical and Life Sciences, Graduate School of Science, Hiroshima University, 1-3-1 Kagamiyama, Higashi-Hiroshima, Hiroshima 739-8526, Japan; 3 Laboratory of Animal Health, Department of Animal Science, Faculty of Agriculture, Tokyo University of Agriculture, 1737 Funako, Atsugi, Kanagawa 243-0034, Japan; George Mason University, UNITED STATES

## Abstract

DNA-editing technology has made it possible to rewrite genetic information in living cells. Human immunodeficiency virus (HIV) provirus, an integrated form of viral complementary DNA in host chromosomes, could be a potential target for this technology. We recently reported that HIV proviral DNA could be excised from the chromosomal DNA of HIV-based lentiviral DNA-transduced T cells after multiple introductions of a clustered regularly interspaced short palindromic repeat (CRISPR)/Cas9 endonuclease system targeting HIV long terminal repeats (LTR). Here, we generated a more efficient strategy that enables the excision of HIV proviral DNA using customized transcription activator-like effector nucleases (TALENs) targeting the same HIV LTR site. A single transfection of TALEN-encoding mRNA, prepared from *in vitro* transcription, resulted in more than 80% of lentiviral vector DNA being successfully removed from the T cell lines. Furthermore, we developed a lentiviral vector system that takes advantage of the efficient proviral excision with TALENs and permits the simple selection of gene-transduced and excised cells in T cell lines.

## Introduction

Three genome-editing technologies that modulate cellular genomic information have become increasingly utilized in recent years. Rapid amelioration of zinc finger nucleases (ZFNs) [1,2], transcription activator-like effector nucleases (TALENs) [3–5], and clustered regularly interspaced short palindromic repeat (CRISPR) [6,7] systems have been developed with high expectations [8–10]. ZFNs and TALENs are programmable nucleases that consist of a zinc finger and transcription activator-like effector, respectively, as a DNA-binding module fused with a non-specific DNA cleavage domain of the restriction nuclease *Fok*I as a DNA cleavage module [11]. The CRISPR-associated endonuclease Cas9 (CRISPR/Cas9), derived from the *Streptococcus pyogenes*, can cleave specific DNA sequences when paired with a complementary non-coding guide RNA (gRNA) [6,12].

All three systems introduce a DNA double-stranded break (DSB) at the target site in living mammalian cells [2], which is typically repaired through major DNA repair mechanisms such as non-homologous end joining (NHEJ) or homology-directed repair (HDR) pathways. The NHEJ is an error-prone repair pathway in which DSBs are re-ligated, leaving behind insertion/deletion (indel) mutations. Therefore, these genome-editing tools are utilized to generate specific gene knockouts [13–16].

Each of the genome-editing technologies has advantages and disadvantages. The CRISPR/Cas9 endonuclease system is endowed with a simplified design and fast handling because the target sequence is directed by complementary gRNA that can be easily synthesized. Thus, the CRISPR/Cas9 system has been employed by numerous groups who have rapidly accelerated the technology, leading to a myriad of reports in a relatively short span of time [6,12,16,17]. However, some groups have reported concerns of off-target cleavage of the CRISPR/Cas9 endonuclease [18,19]. On the other hand, TALENs possess significant precision for the target sequence upon modification [20]. There are some hurdles such as cost, complex design, and time-consuming handling involved in order to construct highly specific TALENs. However, Sakuma *et al*. recently developed a new, customized version of the TALEN system, designated as Platinum TALENs, in which a variant harboring a non-repeat-variable di-residue (non-RVD) exhibits highly efficient genome editing activity that permits its straightforward design and construction [21]. This new TALEN version is highly promising for future utilization.

A crucial step in the replication of human immunodeficiency virus (HIV) occurs when its complementary DNA is integrated into the host genome [22]. The proviral DNA often becomes transcriptionally inactive in CD4+ memory T cells, and serves as a viral reservoir in HIV infected individuals [23]. While current anti-retroviral therapy (ART), consisting of a cocktail of inhibitors targeting several viral enzymes, potently suppresses active virus replication, it is ineffective against latent HIV provirus [24]. The existence of integrated proviruses makes curing HIV disease nearly impossible and, therefore, a new strategy for eliminating the latent form of HIV DNA in HIV-infected individuals needs to be addressed. Since the nucleotide sequence of the HIV provirus is distinct from that of the host genome, the provirus could serve as an appropriate target for genome-editing technologies. We, as well as Qu *et al*., have reported that ZFNs and CRISPR/Cas9 endonucleases targeting HIV LTR could disrupt the function of latent HIV provirus [25,26]. Importantly, because HIV proviral DNA contains duplicate LTR sequences on both ends, it is susceptible to simultaneous cleavage and possible excision by genome-editing technologies, potentially ridding the host genome of the foreign intruder.

In this study, we created new TALENs targeting the TAR region of HIV LTRs (TAR TALENs) to achieve highly efficient editing of HIV proviral DNA. The TAR TALENs showed outstanding editing and excision potential for HIV proviral DNA, particularly via the delivery of mRNAs-encoding TAR TALENs. This excision activity is applicable to remove HIV-based lentiviral vector DNA in T cell lines.

## Materials and Methods

### Ethics statement

This work was reviewed and approved by the Kyoto University institutional review board. Informed written consent from human subjects was obtained.

### Construction of TALENs targeting the HIV-TAR region

FLAG-tagged TALEN plasmids were constructed with the two-step Golden Gate cloning method using a Platinum Gate TALEN Kit (Addgene; cat#1000000043) as previously described [21]. A ptCMV-153/47-VR-NI vector (Addgene; Plasmid 50705) was used for the destination vector. Left and right TALEN target sequences and assembled RVD modules are shown in S1 Table.

### Construction of lentiviral vector DNA

To construct a FLAG-tagged truncated mutant-CPSF6 and enhanced green fluorescent protein (GFP) dual-expressing lentiviral vector DNA, pCSII-EF-CPSF6ΔNC-Flag-IRES-EGFP, DNA fragments encoding the 233–361 amino acid residues of CPSF6 (NM_007007) followed by a FLAG peptide were inserted at the XhoI-EcoRI site of pCSII-EF-MCS-IRES-EGFP [27,28].

To construct a C-C chemokine receptor type 5 (CCR5), herpes simplex virus type 1 (HSV-1) thymidine kinase (TK), and puromycin resistant (Puro) proteins triple expressing a lentiviral vector DNA, pCSII-EF-CCR5-IRES-TK-2A-Puro, custom DNA fragments of HSV-1 *UL23* (Gene ID: 2703374) and self-cleaving 2A peptide (2A)-Puro from PB514B-2 (System Biosciences) were inserted at the XhoI-NotI site of pCSII-EF-MCS-IRES-EGFP via the Gibson assembly cloning kit (New England Biolab). Then *CCR5* complementary DNA (Gene ID: MN_000579) was inserted at the XhoI site in the multi-cloning site of pCSII-EF-MCS-IRES-TK-2A-Puro.

### Cell culture and drug selection

293T cells (RIKEN BRC: RCB2202) were maintained in Dulbecco’s Modified Eagle Medium (DMEM) containing 10% fetal calf serum (FCS), 100 U/ml of penicillin, and 100 μg/ml of streptomycin. Jurkat (RIKEN BRC: RCB0806), c19 [26], MT-4 [29], and these lentiviral vector-transduced and ACH-2 [30] cells were maintained in an RPMI 1640 medium containing 10% FCS, 100 U/ml of penicillin, and 100 μg/ml of streptomycin. To select the cells transduced with the TK-2A-Puro-expressing lentiviral vector, cells were cultured in the presence of 0.5 μg/ml of puromycin (InvivoGen). To displace the cells transduced with TK and Puro dual-expressing lentiviral vectors, cells were cultured in the presence of 10 μg/ml of ganciclovir (GCV) (Abcam). To activate latently integrated provirus in c19, cells were cultured in the presence of 10 ng/ml TNF-α (R&D systems) for 48 hours.

### Transfection (TF) of plasmid DNA and mRNA

T cell lines were transfected by the Neon Transfection System (Life Technologies) in 10 μl tips under the following conditions: 10 ms/Pulse 3/1325 V for parental and lentiviral vector-transduced Jurkat cells, 40 ms/Pulse 1/1230 V for parental and lentiviral vector-transduced MT-4 cells and ACH-2 cells. For TF of the CRISPR/Cas9 system, 1 μg of humanized Cas9 expression DNA and 1 μg of gRNA expression DNA (Addgene) were used. For TF of the TALEN system, 1 μg each of TALEN-L and -R plasmids were used. mRNAs encoding TALENs (mTALENs) and GFP were generated by *in vitro* transcription using the mMESSAGE mMACHINE T7 ULTRA kit (Life Technologies). mRNAs were purified using the MEGAclear kit (Life Technologies) and eluted in RNase-free water. Half a microgram each of TALEN-L and -R mRNAs were used for TF.

### HIV and HIV-based lentiviral vector preparation and infection

To prepare HIV suspensions of NL4–3 and JR-CSF, 293T cells were transfected with 30 μg of either pNL4–3 or pJR-CSF by the calcium-phosphate method and the culture supernatants were collected as previously described [26]. Infectivity of the virus suspensions was titrated in phytohemagglutinin-stimulated human peripheral blood mono nuclear cells (PHA-PBMC), and 50% tissue culture infective doses (TCID_50_) were calculated according to the Reed—Muench method as described [31].

Lentiviral vectors suspensions were prepared as described previously [27,32]. 293T cells were co-transfected with pEV731 (kindly provided by Dr. Eric Verdin), pCS-CDF-CG-PRE, pCS-MCS-TK-2A-Puro, or pCS-CCR5-TK-2A-Puro together with a mixture of helper plasmids, MD.G, pMDLg/pRRE, and pRSV Rev and cultured another 48 hours. The culture supernatants were filtrated through a membrane (pore size 0.45 μm) as described previously [27,28]. The infectivity was measured as described before [28].

To measure multiple-rounds of HIV-1 replication, lentiviral vector-transduced Jurkat cells were infected with NL4–3 or JR-CSF at a MOI of 0.01 and 0.1, respectively, and then the culture supernatants were harvested. The level of HIV-1 p24 antigen was measured by enzyme-linked immunosorbent assay (ELISA) (ZeptoMetrix).

### Flow cytometry

Flow cytometry was performed with a FACSCalibur and a FACSCant II (BD Biosciences) as previously described [26,32], and the data were analyzed using CellQuest software (BD Biosciences) and FlowJo software (Tree Star, Inc.). For detection of internal p24 expression and CCR5 on the cell surface, a fluorescent isothiocyanate (FITC)-conjugated anti-p24 mouse monoclonal antibody (MAb) (CLONE KC57) (Beckman Coulter, Inc.) and phycoerythrin (PE)-conjugated anti-CCR5 (CD195) mouse MAb (BD Biosciences) were used, respectively. For sorting GFP positive cells, FACSAria (BD Biosciences) was used.

### Polymerase chain reaction (PCR) and sequencing

To confirm mutations in the TAR region, a PCR primer set, HE418 and HE414, was used. The PCR products were cloned into a pGEM-T vector (Promega) and sequenced as described previously [26].

To show the length of the HIV provirus in Jurkat c19 cells, a PCR primer set, HE433 and HE435, which is designed for the host cell genome sequence flanking the proviral integration site, was used as described previously [26].

### Western blotting

Western blotting to detect FLAG or tubulin in cells was performed as described previously [33]. Briefly, MT-4 cells were lysed in SDS buffer [34], resolved by 8% SDS-PAGE gels, and transferred to Immobilion P Transfer Membrane (Millipore). Primary antibodies were anti-tubulin mouse MAb (DM1A, Sigma) and anti-FLAG mouse MAb (M2, Invitrogen). Horseradish peroxidase (HRP)- conjugated anti-mouse IgG (Cell Signaling) was used as a secondary antibody.

### Quantification of HIV provirus DNA

The amount of *EGFP* DNA was quantified by real-time PCR as described previously [35,36]. Briefly, genomic DNA was extracted from cells with a DNeasy Blood & Tissue Kit (QIAGEN). Real-time PCR was performed with Sybr Green using primer sets specific for EGFP [35] and b-actin [36]. Relative levels of EGFP DNA were measured based on the obtained value of EGFP DNA per b-actin DNA.

### Statistical analysis

The data for statistical analyses are averages of three independent experiments. All data were expressed as mean ± standard deviations (S.D.). The significance of differences in the means was determined by the Student's t test. *P* values are shown in each figure.

## Results

### Efficient mutation and disruption of HIV LTR by new TALENs targeting LTR

**Fig 1:**
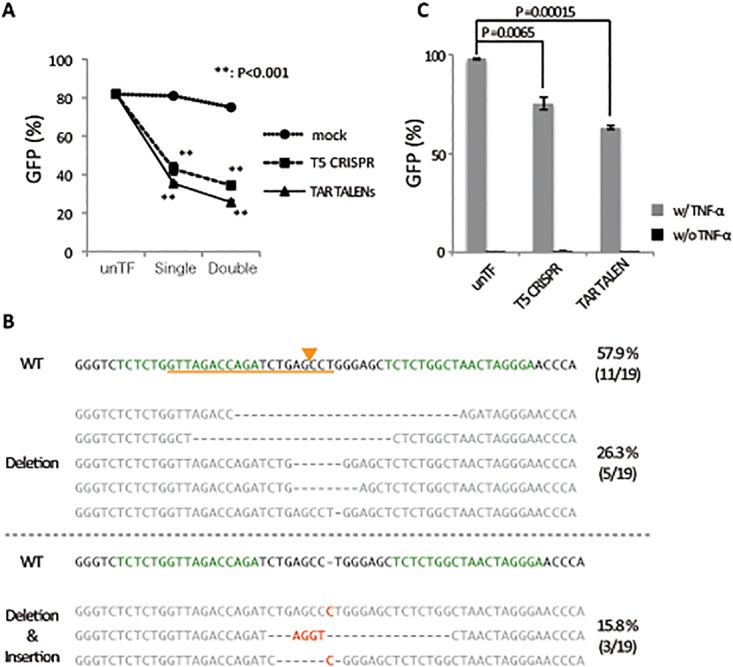
Genome-editing activity of the TALENs and CRISPR/Cas9 systems targeting HIV LTR. (A) LTR-driven GFP expression after TF with CRISPR/Cas9 or TALENs expressing plasmid DNA. Jurkat cells were infected with an LTIG lentiviral vector. Five days after infection, the GFP positive cells were sorted (Jurkat/LTIG) and co-transfected with T5 gRNA-expressing and hCas9-expressing DNA or LTR TALEN-L and -R expressing DNA. The level of GFP expression was analyzed by flow cytometry 5 days after TF. (B) Sequence analysis of TALENs targeting sites. The DNA sequences of the TAR and adjacent regions of LTR are indicated. Nineteen sequences were obtained from Jurkat/LTIG cells, which were transfected twice with TAR TALEN-LR. The WT reference sequence is shown at the top. The target sequences of TAR TALENs and T5 gRNA are indicated in orange and green, respectively. The putative cleavage site of T5 CRISPR is indicated with an orange arrowhead. (C) Genome-editing activity of TAR TALENs and T5 CRISPR in c19 cells, latently transduced with an LTIG lentiviral vector. The level of GFP expression after 48 hours of TNF-α stimulation is shown. The error bars in B and D show standard deviations (n = 3).

Previous genome editing studies using ZFNs and CRISPR/Cas9 systems indicated that HIV LTR is a prominent target for mutation-inducing disruption of HIV provirus function [25,26]. To improve the targeting of the HIV LTR sequence for genome-editing technology, we used a new TALEN system, Platinum TALENs, that is modified with periodically patterned repeats harboring non-RVD variations and possesses extremely high cleavage activity [21]. We used TALENs to target the same TAR region of HIV LTR, which is proven to be critical for gene expression in HIV proviral DNA and susceptible to CRISPR/Cas9 nuclease activity. Initially, we evaluated the LTR-editing activity in T cells. Jurkat cells transduced with an LTIG lentiviral vector (Jurkat/LTIG), which expresses both Tat and GFP proteins under the control of LTR and mimics authentic HIV gene expression, were transfected with the TAR TALENs DNA or Cas9 DNA together with gRNA targeting TAR (T5 CRISPR), and the LTR-driven GFP expression was analyzed by flow cytometry (Fig. 1A). As previously reported, single and double TF of T5 CRISPR led to a significant decrease in the average percentage of GFP positive cells from 81.9% to 43.0% (*p* = 0.002) and to 34.5% (*p* = 0.000001), respectively. It is noteworthy that single and double TF of the TAR TALENs decreased the average percentage of GFP positive cells even more significantly from 81.9% to 35.5% (*p* = 0.000002) and to 25.7% (*p* = 0.0003), respectively. We confirmed a variety of indel mutations in the LTR sequence by double TF of the TAR TALENs (Fig. 1B), as previously observed with T5 CRISPR [26]. Although TAR TALENs showed sufficient HIV proviral-editing activity, we were concerned about the editing ability of TALENs to target latent HIV provirus. To examine this, an LTIG-transduced Jurkat cell clone, c19 [26], which possesses one copy of the latent HIV provirus on chromosome 16, which can be induced after TNF-α stimulation, was transfected with TAR TALENs or T5 CRISPR plasmid DNA. Four days later, cells were stimulated with TNF-α, and then GFP-expressing cells were measured by flow cytometry 48 hours after induction (Fig. 1C). By single TF of T5 CRISPR, the average percentage of GFP positive cells was reduced from 97.8% to 75.3% (*p* = 0.0065). In contrast, by a single TF of TAR TALENs, the average percentage of GFP positive cells was significantly decreased to 63.0% (*p* = 0.00015). It is possible that the lower basal transfection efficiency in c19 cells reflected the lower editing efficiency in these cells compared with Jurkat cells transduced with LTIG vector (Fig. 1A and C). These results indicated that the newly generated TAR TALENs are a powerful tool for HIV proviral editing and appeared to be more efficient than the T5 CRISPR for disrupting the HIV LTR function.

### Disruption potential of TAR TALENs mRNAs for HIV LTR function in T cells

**Fig 2:**
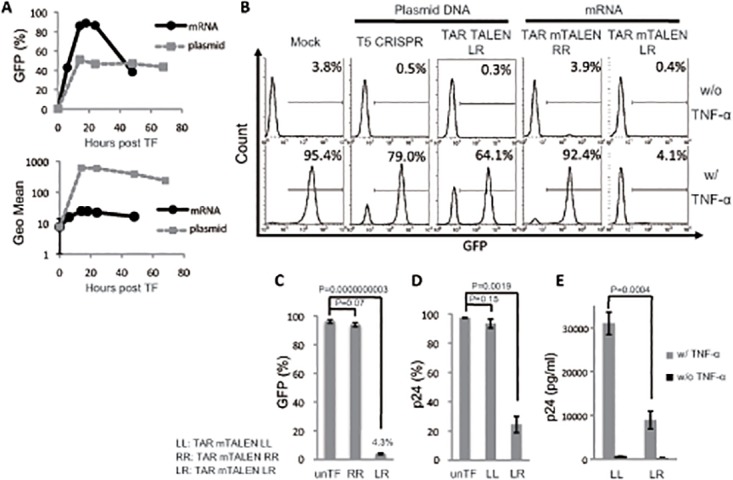
HIV LTR editing with mRNAs of TAR TALENs. (A) Kinetics of GFP transduction with mRNA and plasmid DNA in Jurkat cells. Jurkat cells were transfected with 1 μg of mRNA GFP or 1 μg of plasmid GFP under the control of a CMV promoter. The time course analysis of GFP expression was performed by flow cytometry. (B and C) A Jurkat cell line latently transduced with an LTIG vector was transfected with mTALENs. The level of GFP expression 48 hours after TNF-α stimulation is shown. Representative histograms are shown in B. The positive percentage of GFP is shown in B (n = 3). (D and E) ACH-2 cells were transfected with TAR mTALENs. The expression of p24 antigen 48 hours after TNF-α stimulation is shown. The percentage of p24 antigen expression in cells is shown in C (n = 3). The amount of p24 antigen in the culture supernatant is shown in E (n = 3). The error bars in A, C, D, and E show standard deviations (n = 3).

To improve the efficiency of TALENs transduction in T cells, we examined another TF strategy using mRNA synthesized by *in vitro* transcription. Initially, mRNA encoding GFP (mRNA GFP) was used to optimize TF conditions in T cell lines. Jurkat cells were transfected with 1 μg of mRNA GFP or plasmid DNA encoding GFP (plasmid GFP), and the kinetics of GFP expression were analyzed. A significantly higher percentage of GFP positive cells was obtained with TF of mRNA GFP than with TF of plasmid GFP (Fig. 2A, left panel), while the maximum mean fluorescence intensity and the duration of GFP expression after mRNA GFP TF were limited (Fig. 2A, right panel). This result suggested that the TF of mRNAs encoding TALENs might improve the frequency of transduction and increase the likelihood of genome editing in T cells. Next, we synthesized mRNA of FLAG-tagged TALENs targeting HIV-1 TAR (TAR mTALEN-L and TAR mTALEN-R) by *in vitro* transcription and confirmed protein expression in Jurkat cells transfected with TAR mTALENs (S1A Fig.). Then, the effects of TAR mTALENs on HIV proviral editing was assessed in c19 cells as shown in Fig. 1C. Combinational TF of TAR mTALEN-L and -R (shown as LR) drastically suppressed the mean percentage of GFP positive cells after stimulation with TNF-α (Fig. 2B and C). Single TF of TAR mTALEN-LR led to a profound suppression in the mean percentage of GFP positive cells to 4.3%, while it was 63.0% in the cells transfected with plasmid DNA expressing the TAR TALENs (Fig. 2C and 1C). The suppression of proviral reactivation was not observed in the cells transfected with TAR mTALEN-R alone (92.23%, shown as RR).

To list potential off-target sites for TAR TALENs in chromosomal cellular DNA, we used the paired target finder tool TAL Effector Nucleotide Targeter 2.0 [37]. All listed potential off-target sites showed more than a 12-nucleotide difference in the TAL binding sequence (S2 Table). We performed sequencing analysis for one of the potential off-target sites in the c19 cells treated with TAR mTALEN-LR (shown in Fig. 2B); however, not a single mutation was detected in 50 clones sequenced (S3 Table). These results suggested that TAR TALENs had no detectable off-target effects.

To confirm the activity of TAR mTALENs, another cell line, ACH-2 cells, was examined. The ACH-2 cell line is a subclone of T cells persistently infected with HIV-1_LAV_ [30]. The cells harbor the proviral DNA but, express and produce limited levels of viral proteins, which can be induced by supplementing cells with TNF-α [30]. ACH-2 cells were transfected with TAR mTALEN-LR or TAR mTALEN-LL. Four days later, the cells were stimulated with TNF-α. The mean percentage of Gag p24 positive cells was 24.5% in TAR mTALEN-LR-treated cells, and the reduction was significant compared with either untransfected or TAR mTALEN-L-alone transfected cells (Fig. 2D, *p* = 0.0003). The level of released viral proteins from TAR mTALEN-LL- or TAR mTALEN-LR-transfected cells was measured after 2 days of stimulation with TNF-α by p24 ELISA (Fig. 2E). We confirmed a significant reduction of the Gag p24 antigen in the supernatant of TAR mTALEN-LR-transfected cells (3.47 fold reduction, *p* = 0.0004). These results suggested that the alternative transfection strategy using TAR mTALENs drastically improved the disruption efficiency of the HIV proviral function in T cell lines.

### Robust excision potential of TAR mTALENs for HIV provirus

**Fig 3:**
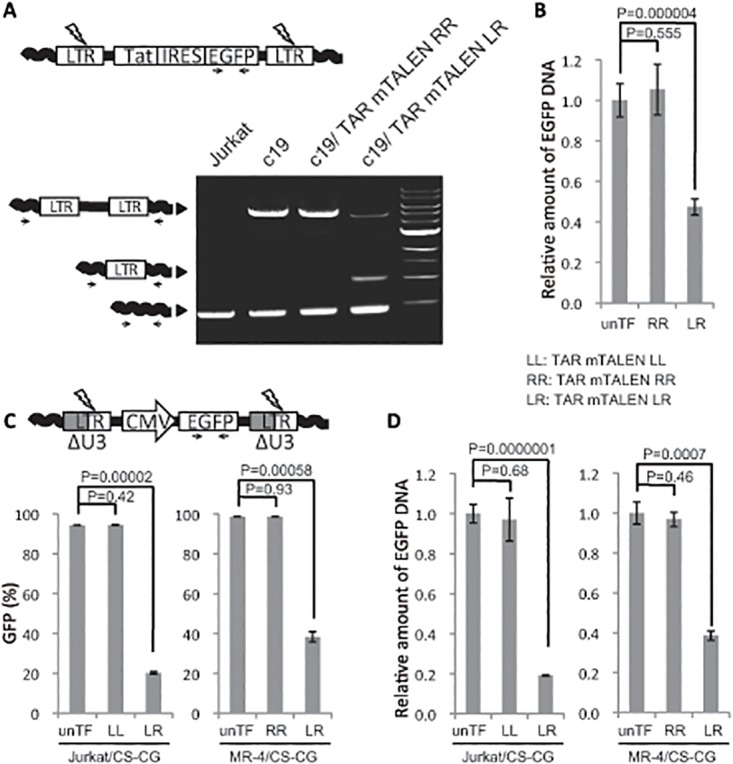
Excision of HIV provirus from host cell genome with TAR mTALENs. (A and B) HIV proviral excision in c19. (A) HIV provirus in c19 treated with TAR mTALENs was amplified using a primer set designed for the host cell genome sequence flanking the proviral integration site. The schematic of PCR products indicating genomic sequences, full-length provirus, and one LTR footprint resulting from proviral excision are shown on the left side. (B) The relative amount of *EGFP* DNA in TAR mTALENs—treated c19 is shown. (C and D) Excision of HIV-based lentiviral vector DNA with TAR mTALENs. Jurkat and MT-4 cells were transduced with a lentivirus vector containing a CMV promoter—derived GFP-expressing cassette, Jurkat/CS-CG and MT-4/CS-CG cells, respectively, and, these cells were treated with TAR mTALENs. A schematic of the lentiviral vector DNA used in this assay is shown at the top of C. The percentage of GFP positive cells after TF of mTALENs is shown in C (n = 3). The relative amount of *EGFP* DNA in treated TAR mTALENs is shown in D (n = 3). The error bars in B, C, and D show standard deviations (n = 3).

We recently reported that LTR cleavage with T5 CRISPR could lead to the excision of HIV proviral DNA [26]. We assumed that efficient LTR cleavage with TAR mTALENs could excise proviral DNA more efficiently. To examine this possibility, proviral DNA in the cells shown in Fig. 2C was assessed by PCR using a primer set specific to the adjacent sequences for both ends of the integrated LTR of the provirus in c19 cells as described previously [26]. As expected, a significant number of one LTR footprint that is generated only from the excision of proviral DNA was detected in cells treated with TAR mTALEN-LR but not with TAR mTALEN-RR (Fig. 3A). Quantitative PCR analysis of *EGFP* revealed that a one-shot TF of LTR mTALEN-LR successfully removed more than half of the *EGFP* gene encoded from the integrated form of the lentiviral vector DNA (Fig. 3B, mean removal percentage:53%, *p* = 0.000004).

To assess the possibility of TAR mTALENs being utilized as a therapeutic tool, we tested the anti-HIV effect in primary T cells. PHA-PBMC was transduced with LTIG vector and subsequently transfected with TAR mTALENs. Then, the level of GFP expression was analyzed by flow cytometry. Although the level of GFP positive cells slightly, but not significantly, decreased after TF with TAR mTALEN-LR, no indel mutations at the TAR region were found in the TAR mTALEN-LR transfected PHA-PBMC culture (S2B and S2C Fig.). To examine the possible toxic side effects of TAR TALENs in primary cells, Trypan blue staining was performed. Cell viability two days post transfection was comparable in the mTALEN-LR and -RR transfected cultures (S2D Fig.). Furthermore, no indel mutations were detected even at early timepoints of TAR mTALENs transfection (S2E Fig.). These results suggested that the ineffectiveness of TAR TALENs in primary T cells might not be related to its toxicity. We then performed western blot analysis to examine the level of TALEN proteins in primary T cells. The result showed that only modest TALEN expression was detected in the TAR mTALEN-LR transfected T cells (S2F Fig.). It has been reported that delivery of the TALENs as a DNA species mediated higher rates of editing compared with mRNA in primary fibroblasts [38]. Hence, an explanation of the poor editing activity we observed might be attributed to mRNA being an incompatible vehicle for TALEN transduction in primary T cells.

### Excision system of HIV-based lentiviral vector from the transduced cells

We next examined the possibility of using the TAR mTALENs system for removing transgenes derived from an HIV-based lentiviral vector in cell culture. We assessed the excision ability of TAR mTALENs in Jurkat and MT-4 cells. A self-inactivating (SIN) HIV-based lentiviral vector missing part of the U3 region of the LTR and possessing an internal cytomegalovirus (CMV) promoter cassette for GFP expression was used for the assay (Fig. 3C) [28]. In SIN vector-transduced cells, the population of the GFP negative converted cells represents successful proviral excision. One-shot TF of TAR mTALEN-LR resulted in a significant decrease in the average of GFP positive cells from 94.3% to 20.1% and from 98.7% to 38.3% in Jurkat/CS-CG and MT-4/CS-CG cells, respectively (Fig. 3C and D; *p* value was 0.00002 and 0.0000001, respectively). The quantitative PCR results showed a similar trend in which the average removal of the *EGFP* gene in Jurkat/CS-CG and MT-4/CS-CG cells was 80.9% and 61.4%, respectively (Fig. 3C and D; *p* values were 0.00058 and 0.0007, respectively). These results suggested that TAR mTALENs possess robust excision potential for lentiviral DNA in T cell lines.

**Fig 4:**
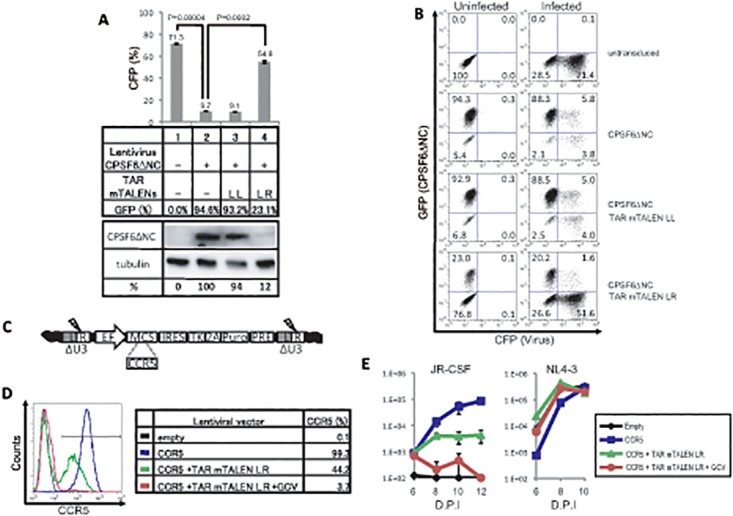
Excision of HIV-based lentiviral vector with TAR mTALENs. (A and B) Excision of lentivirus vector expressing CPSF6ΔNC and GFP with TAR mTALENs and the HIV susceptibility of the cells. MT-4 cells, transduced with a lentivirus vector expressing CPSF6ΔNC and GFP, was treated with TAR mTALENs. Then the cells were infected with an HIV-based lentiviral vector expressing CFP as indicated in the middle panel of A. The percentage of CFP positive cells is shown in the upper panel of A (n = 3). The FLAG-tagged CPSF6ΔNC expressed from the lentivirus vector and the internal expression of tubulin were detected by anti-FLAG and anti-tubulin antibodies, respectively, as shown in the lower panel of A. The original picture of the membranes is shown in S1 Fig. Representative flow cytometry profiles are shown in B. (C) Schematic of developed lentiviral vector DNA. CCR5 genes were inserted into the MCS. (D) Flow cytometry analysis of CCR5 expressed on cells transduced by the lentiviral vector. (E) Multiple-round HIV-1 replication after challenging the indicated cell with two HIV-1 strains, NL4–3 and JR-CSF. HIV production was monitored by measuring the p24 antigen in culture supernatants. The error bars in A and F show standard deviations (n = 3).

To assess the practical applications of TAR TALENs for removing the transgene derived from HIV-based lentiviral vectors in cell culture, a truncation mutant of cleavage and polyadenylation specific factor 6 (CPSF6ΔNC), a host factor restricting HIV infection [39], was initially chosen as a transgene. Then, we attempted to expropriate restriction of HIV infection with CPSF6ΔNC using TAR mTALENs (Fig. 4A). The MT-4 cells transduced with CPSF6ΔNCs and the GFP dual-expressing lentiviral vector were assessed for HIV susceptibility using a VSV pseudotyped CFP-expressing lentiviral vector. CPSF6ΔNC transduction inhibited lentiviral infection by 86.5% (from 71.3 ± 0.70% to 9.7 ± 0.06%). When the CPSF6ΔNC-transduced cells were treated with the TAR mTALENs, the average percentage of GFP (CPSF6ΔNC) positive cells significantly decreased from 94.6% to 23.1%, and the mean percentage of CFP positive cells, lentiviral infected cells, was restored from 9.7% to 54.6% (Fig. 4A and B). We also confirmed that the mean percentage of FLAG-tagged CPSF6ΔNC expression with the lentiviral vector in MT-4 cells was decreased to 12% after single TF of TAR mTALENs (Fig. 4A, bottom).

Using the efficient excision property of the TAR mTALENs, we developed an additional lentiviral vector system. The lentivirus vector was constructed to express a particular gene of interest together with Puro and HSV-1 TK proteins, to facilitate the selection of the gene transduced or excised cells by supplementing puromycin or GCV, respectively (Fig. 4C). To examine the effectiveness of this strategy, we transduced and excised the *CCR5* gene that encodes the co-receptor of R5 tropic HIV. Jurkat cells, which endogenously express both CD4 and CXCR4 and therefore are susceptible to X4 tropic HIV, were infected with a CCR5-expressing lentiviral vector and the CCR5-transduced cells were enriched with puromycin (CCR5 cells). A portion of the CCR5+ cells was treated with TAR mTALENs and then cultured in the absence (CCR5+TAR-mTALENs cells) or presence of GCV (CCR5+TAR-mTALENs +GCV cells). The level of CCR5 surface expression on these cells was analyzed by flow cytometry (Fig. 4D). As expected, almost all cells transduced and then selected by puromycin expressed CCR5 (average percent expression 99.3%), and the expression was clearly reduced up to 44.2% after single TF of TAR mTALENs. Furthermore, only few cells expressed CCR5 after subsequent GCV treatment (average percent expression 3.7%). These cells were challenged with R5 tropic- and X4 tropic-HIV, JR-CSF and NL4–3, respectively, and the amount of p24 antigen in the culture supernatant was measured by ELISA (Fig. 4E). A significant increase in p24 production was detected from CCR5-transduced Jurkat cells after infection with R5 tropic HIV-1 (JR-CSF) over time. On the other hand, a lower level of p24 production was detected from the CCR5+ TAR mTALENs cells that express CCR5 in around half of the population. Importantly, no increase on p24 production was observed from the CCR5 +TAR mTALENs +GCV cells after R5 tropic virus infection. All cells maintained a high level of susceptibility to the X4 tropic virus (NL4–3). These results indicated that the TAR mTALENs system is applicable for efficient HIV-based lentiviral excision from the transduced T cells, and can be utilized as a transgene on/off system when combined with the new lentiviral vector system.

## Discussion

In this study, we successfully edited more than 90% of the HIV provirus in T cell lines upon the single introduction of a DNA-editing system. One of the reasons for the high success rate was because we designed and generated highly potent TALE nucleases targeting HIV provirus using the Platinum TALENs. The Platinum TALEN platform has robust nuclease activity [21]. The enhanced cleavage potential of the new version of TALENs may provide extremely efficient editing activity for HIV provirus. Another reason for the robust activity we observed in the T cell lines is that we found a sufficient method for the delivery of Platinum TALENs. In this study, the disruption efficiency of the HIV proviral genes with TAR TALENs was dramatically improved by using the mRNAs of TAR TALENs. The comparison of TF with mRNA GFP and plasmid GFP showed that the mRNA TF produces a remarkable frequency of transduction, though it is clearly limited in the level and duration of expression in transduced cells (Fig. 2A). It has been reported that an improved efficiency of genome-editing activity is observed in cells expressing a higher level of ZFNs or Cas9 nucleases compared with cells expressing a lower level of these nucleases [40]. It is also possible that excessive amounts of nucleases are unnecessary for a sufficient level of editing in the case of highly proficient nucleases such as the TAR TALENs.

In this study, we could not detect any off-target effects by TAR TALENs (S3 Table). Claudio *et al*. reported that TALENs showed less off-target effects compared with ZFNs [20]. They obtained these results using the TALENs without non-RVD variations. On the other hand, the TAR TALENs used in this study harbor non-RVD variations and resulted in high genome-editing activity (Fig. 1A). Because the target specificity of TAR TALENs might also be higher than that of the TALENs without non-RVD, they may possess lower off-target frequency. In addition, the mRNA TF method limited the gene expression both in the amount and the duration compared to plasmid DNA TF (Fig. 2A and S1 Fig.), meaning that this method may reduce off-target effects resulting from excessive levels of TALE nucleases typically obtained via transduction.

It is reported that the efficiency of gene knockouts with ZFNs is augmented by activating the chromatin status of the target site [41]. Therefore, we initially thought that there might be a difference in target accessibility between CRISPR/Cas9 and the TALENs systems, depending on the proviral integration sites. However, we could not observe any noticeable differences in HIV provirus editing by CRISPR/Cas9 and TALENs, even when proviruses were widely distributed around the whole genome (Fig. 1A). Previous reports have shown that HIV preferentially integrates its complementary DNA into active gene regions [42–44]. The integration site preferences of active genes might contribute to the efficient proviral editing in both CRISPR/Cas9 or TALENs systems, which indicates that the HIV provirus is an appropriate target for genome-editing technology. Moreover, our newly devised lentiviral vector system enabled gene transduction and efficient transgene excision when combined with the TAR mTALENs system (Fig. 4). The new transgene IN/OUT system based on the remarkable compatibility of the lentiviral vector and TAR TALENs may serve as a useful tool for discriminating epigenetic alternation induced by effects of its transgene or integration in T cells after lentivirus transduction.

Unfortunately, the TAR mTALENs did not show clear anti-HIV activity in primary human T cells. Initially, we hypothesized that the low activity of TAR TALENs in primary T cells resulted from toxic effects, but cell viability was comparable in the mTALEN-LR and -RR transduced cells (S2D Fig.). We also ruled out the possibility that the NHEJ repair activity was low because a recent study showed that genome-editing activities were observed in primary T cells introduced with CRISPR or ZFNs [45,46]. Incidentally, an insufficient level of TALEN protein expression may explain the low activity of TALENs in primary T cells. We detected only a modest expression of TALENs in primary T cells when TAR mTALENs were transfected (S2F Fig.). Interestingly, it was reported that the delivery of TALENs as a DNA species induced higher rates of editing compared with mRNA in primary fibroblast [38]. This is in contrast to our results observed in Jurkat cells, and suggested that mRNA may not be a suitable vehicle for TALENs transduction in primary T cells.

The TAR TALENs has great potential to be a therapeutic tool for anti-HIV therapy because of its robust genome-editing activity with minimal off-target effects. Hence, future efforts will focus on other strategies for TAR TALENs delivery, such as an adeno-associated virus (AAV) vector because it may be useful for evaluating the availability and applicability of the TAR TALENs in primary T cells for therapeutic purposes.

## Supporting Information

S1 Fig Expression of TALEN proteins in Jurkat cells and time course analysis of GFP expression in c19 treated with TNF-α. (A) TALENs expression in Jurkat cells. Jurkat cells were transfected with plasmids and mRNAs encoding TAR TALENs. The level of protein expression was detected with anti-FLAG antibody. (B) Time course analysis of GFP expression in c19 treated with TNF-α. The level of GFP expression in c19 was analyzed by flow cytometry after 18, 24. 48, and 72 hours post TNF-α stimulation. The positive percentage of GFP (upper panel) and MFI (lower panel) are shown (n = 3).
(EPS)

S2 Fig Genome editing activity of TAR TALENs in primary human T cells. (A) TF efficiency of mRNA in human primary T cells. PHA-PBMC was transfected with 1 μg of mRNA or plasmid DNA encoding GFP. The GFP positive cells were analyzed by flow cytometry after 72 hours. Neon transfection was performed in 10 μl tips under the following condition; 15 ms/Pulse 2/1415 V. (B) Transduction of TAR TALENs modestly decreased GFP positive cells. PHA-PBMCs were infected with LTIG lentiviral vector. Two days after LTIG infection, the cells were transfected with mRNAs of TAR TALENs using the Neon system. The population of GFP positive cells was analyzed by flow cytometry at 10 days after TF. (C) Sequence analysis of TAR TALENs targeting sites. The DNA sequences of the TAR and adjacent regions of LTR are indicated. Forty and twenty sequences of TAR regions were obtained from PHA-PBMC/LTIG generated by donor A and B respectively. No indel mutations were detected in a total of 60 sequenced clones. (D, E, F) Primary T cells were transfected with TAR mTALENs and then cultured for another two days. Trypan Blue staining for cell viability (D), sequencing of target region (E), western blot analysis to show the level of TALENs expression (F) were performed. The error bars in A, B and D show standard deviations (n = 3).
(EPS)

S1 Table Information of TAR TALENs. Target sequence and RVD sequence of TAR TALEN-L and -R are indicated.
(XLSX)

S2 Table Potential off-target sites of TAR TALENs. Potential off-target sites of TAR TALENs were predicted by TAL Effector Nucleotide Targeter 2.0.
(XLSX)

S3 Table Sequencing analysis of a potential off-target site in c19 cells treated with TAR mTALEN-LR. Detected sequences at a potential off-target site in TAR mTALENs transduced cells are indicated.
(XLSX)

## References

[1] Urnov FD, Miller JC, Lee YL, Beausejour CM, Rock JM, Augustus S, et al (2005) Highly efficient endogenous human gene correction using designed zinc-finger nucleases. Nature 435: 646–651. 15806097 10.1038/nature03556

[2] Urnov FD, Rebar EJ, Holmes MC, Zhang HS, Gregory PD (2010) Genome editing with engineered zinc finger nucleases. Nat Rev Genet 11: 636–646. 10.1038/nrg2842 20717154

[3] Mahfouz MM, Li L, Shamimuzzaman M, Wibowo A, Fang X, Zhu JK (2011) De novo-engineered transcription activator-like effector (TALE) hybrid nuclease with novel DNA binding specificity creates double-strand breaks. Proc Natl Acad Sci U S A 108: 2623–2628. 10.1073/pnas.1019533108 21262818 PMC3038751

[4] Boch J, Scholze H, Schornack S, Landgraf A, Hahn S, Kay S, et al (2009) Breaking the code of DNA binding specificity of TAL-type III effectors. Science 326: 1509–1512. 10.1126/science.1178811 19933107

[5] Moscou MJ, Bogdanove AJ (2009) A simple cipher governs DNA recognition by TAL effectors. Science 326: 1501 10.1126/science.1178817 19933106

[6] Mali P, Yang L, Esvelt KM, Aach J, Guell M, DiCarlo JE, et al (2013) RNA-guided human genome engineering via Cas9. Science 339: 823–826. 10.1126/science.1232033 23287722 PMC3712628

[7] Sander JD, Joung JK (2014) CRISPR-Cas systems for editing, regulating and targeting genomes. Nat Biotechnol 32: 347–355. 10.1038/nbt.2842 24584096 PMC4022601

[8] Huang P, Xiao A, Zhou M, Zhu Z, Lin S, Zhang B (2011) Heritable gene targeting in zebrafish using customized TALENs. Nat Biotechnol 29: 699–700. 10.1038/nbt.1939 21822242

[9] Wang H, Yang H, Shivalila CS, Dawlaty MM, Cheng AW, Zhang F, et al (2013) One-Step Generation of Mice Carrying Mutations in Multiple Genes by CRISPR/Cas-Mediated Genome Engineering. Cell 153: 910–918. 10.1016/j.cell.2013.04.025 23643243 PMC3969854

[10] Hutter G, Nowak D, Mossner M, Ganepola S, Mussig A, Allers K, et al (2009) Long-term control of HIV by CCR5 Delta32/Delta32 stem-cell transplantation. N Engl J Med 360: 692–698. 10.1056/NEJMoa0802905 19213682

[11] Gaj T, Gersbach CA, Barbas CF 3rd (2013) ZFN, TALEN, and CRISPR/Cas-based methods for genome engineering. Trends Biotechnol 31: 397–405. 10.1016/j.tibtech.2013.04.004 23664777 PMC3694601

[12] Ran FA, Hsu PD, Wright J, Agarwala V, Scott DA, Zhang F (2013) Genome engineering using the CRISPR-Cas9 system. Nat Protoc 8: 2281–2308. 10.1038/nprot.2013.143 24157548 PMC3969860

[13] Perez EE, Wang J, Miller JC, Jouvenot Y, Kim KA, Liu O, et al (2008) Establishment of HIV-1 resistance in CD4+ T cells by genome editing using zinc-finger nucleases. Nat Biotechnol 26: 808–816. 10.1038/nbt1410 18587387 PMC3422503

[14] Holt N, Wang J, Kim K, Friedman G, Wang X, Taupin V, et al (2010) Human hematopoietic stem/progenitor cells modified by zinc-finger nucleases targeted to CCR5 control HIV-1 in vivo. Nat Biotechnol 28: 839–847. 10.1038/nbt.1663 20601939 PMC3080757

[15] Sung YH, Baek IJ, Kim DH, Jeon J, Lee J, Lee K, et al (2013) Knockout mice created by TALEN-mediated gene targeting. Nat Biotechnol 31: 23–24. 10.1038/nbt.2477 23302927

[16] Cong L, Ran FA, Cox D, Lin S, Barretto R, Habib N, et al (2013) Multiplex genome engineering using CRISPR/Cas systems. Science 339: 819–823. 10.1126/science.1231143 23287718 PMC3795411

[17] Mali P, Esvelt KM, Church GM (2013) Cas9 as a versatile tool for engineering biology. Nat Methods 10: 957–963. 10.1038/nmeth.2649 24076990 PMC4051438

[18] Fu Y, Foden JA, Khayter C, Maeder ML, Reyon D, Joung JK, et al (2013) High-frequency off-target mutagenesis induced by CRISPR-Cas nucleases in human cells. Nat Biotechnol 31: 822–826. 10.1038/nbt.2623 23792628 PMC3773023

[19] Cradick TJ, Fine EJ, Antico CJ, Bao G (2013) CRISPR/Cas9 systems targeting beta-globin and CCR5 genes have substantial off-target activity. Nucleic Acids Res 41: 9584–9592. 10.1093/nar/gkt714 23939622 PMC3814385

[20] Mussolino C, Morbitzer R, Lutge F, Dannemann N, Lahaye T, Cathomen T (2011) A novel TALE nuclease scaffold enables high genome editing activity in combination with low toxicity. Nucleic Acids Res 39: 9283–9293. 10.1093/nar/gkr597 21813459 PMC3241638

[21] Sakuma T, Ochiai H, Kaneko T, Mashimo T, Tokumasu D, Sakane Y, et al (2013) Repeating pattern of non-RVD variations in DNA-binding modules enhances TALEN activity. Sci Rep 3: 3379 10.1038/srep03379 24287550 PMC3843162

[22] Craigie R, Bushman FD (2012) HIV DNA Integration. Cold Spring Harb Perspect Med 2: a006890 10.1101/cshperspect.a006890 22762018 PMC3385939

[23] Siliciano RF, Greene WC (2011) HIV latency. Cold Spring Harb Perspect Med 1: a007096 10.1101/cshperspect.a007096 22229121 PMC3234450

[24] Eisele E, Siliciano RF (2012) Redefining the viral reservoirs that prevent HIV-1 eradication. Immunity 37: 377–388. 10.1016/j.immuni.2012.08.010 22999944 PMC3963158

[25] Qu X, Wang P, Ding D, Li L, Wang H, Ma L, et al (2013) Zinc-finger-nucleases mediate specific and efficient excision of HIV-1 proviral DNA from infected and latently infected human T cells. Nucleic Acids Res 41: 7771–7782. 10.1093/nar/gkt571 23804764 PMC3763554

[26] Ebina H, Misawa N, Kanemura Y, Koyanagi Y (2013) Harnessing the CRISPR/Cas9 system to disrupt latent HIV-1 provirus. Sci Rep 3: 2510 10.1038/srep02510 23974631 PMC3752613

[27] Kawano Y, Yoshida T, Hieda K, Aoki J, Miyoshi H, Koyanagi Y (2004) A lentiviral cDNA library employing lambda recombination used to clone an inhibitor of human immunodeficiency virus type 1-induced cell death. J Virol 78: 11352–11359. 15452256 10.1128/JVI.78.20.11352-11359.2004 PMC521860

[28] Miyoshi H, Blomer U, Takahashi M, Gage FH, Verma IM (1998) Development of a self-inactivating lentivirus vector. J Virol 72: 8150–8157. 9733856 10.1128/jvi.72.10.8150-8157.1998 PMC110156

[29] Miyoshi I, Taguchi H, Kubinishi I, Yoshimoto S, Ohtsuki Y, Shiraishi Y, et al (1982) Type C virus-producing cell lines derived from adult T-cell leukemia In: Hanaoka M, Takatsuki K, Shimoyama M, editors. Gann monographs on cancer research. Tokyo: Japan Scientific Societies Press pp. 219–237.

[30] Clouse KA, Powell D, Washington I, Poli G, Strebel K, Farrar W, et al (1989) Monokine regulation of human immunodeficiency virus-1 expression in a chronically infected human T cell clone. J Immunol 142: 431–438. 2463307

[31] Kawano Y, Tanaka Y, Misawa N, Tanaka R, Kira JI, Kimura T, et al (1997) Mutational analysis of human immunodeficiency virus type 1 (HIV-1) accessory genes: requirement of a site in the nef gene for HIV-1 replication in activated CD4+ T cells in vitro and in vivo. J Virol 71: 8456–8466. 9343202 10.1128/jvi.71.11.8456-8466.1997 PMC192308

[32] Ebina H, Kanemura Y, Suzuki Y, Urata K, Misawa N, Koyanagi Y (2012) Integrase-independent HIV-1 infection is augmented under conditions of DNA damage and produces a viral reservoir. Virology 427: 44–50. 10.1016/j.virol.2012.02.004 22374236

[33] Yamamoto SP, Okawa K, Nakano T, Sano K, Ogawa K, Masuda T, et al (2011) Huwe1, a novel cellular interactor of Gag-Pol through integrase binding, negatively influences HIV-1 infectivity. Microbes Infect 13: 339–349. 10.1016/j.micinf.2010.12.002 21167302

[34] Shinoda Y, Hieda K, Koyanagi Y, Suzuki Y (2009) Efficient transduction of cytotoxic and anti-HIV-1 genes by a gene-regulatable lentiviral vector. Virus Genes 39: 165–175. 10.1007/s11262-009-0382-x 19554442

[35] Godbey WT, Zhang X, Chang F (2008) The importance of and a method for including transfection efficiency into real-time PCR data analyses. Biotechnol Bioeng 100: 765–772. 10.1002/bit.21811 18306419

[36] Suzuki Y, Misawa N, Sato C, Ebina H, Masuda T, Yamamoto N, et al (2003) Quantitative analysis of human immunodeficiency virus type 1 DNA dynamics by real-time PCR: integration efficiency in stimulated and unstimulated peripheral blood mononuclear cells. Virus Genes 27: 177–188. 14501196 10.1023/a:1025732728195

[37] Doyle EL, Booher NJ, Standage DS, Voytas DF, Brendel VP, Vandyk JK, et al (2012) TAL Effector-Nucleotide Targeter (TALE-NT) 2.0: tools for TAL effector design and target prediction. Nucleic Acids Res 40: W117–122. 10.1093/nar/gks608 22693217 PMC3394250

[38] Osborn MJ, Starker CG, McElroy AN, Webber BR, Riddle MJ, Xia L, et al (2013) TALEN-based gene correction for epidermolysis bullosa. Mol Ther 21: 1151–1159. 10.1038/mt.2013.56 23546300 PMC3677309

[39] Lee K, Ambrose Z, Martin TD, Oztop I, Mulky A, Julias JG, et al (2010) Flexible use of nuclear import pathways by HIV-1. Cell Host Microbe 7: 221–233. 10.1016/j.chom.2010.02.007 20227665 PMC2841689

[40] Duda K, Lonowski LA, Kofoed-Nielsen M, Ibarra A, Delay CM, Kang Q, et al (2014) High-efficiency genome editing via 2A-coupled co-expression of fluorescent proteins and zinc finger nucleases or CRISPR/Cas9 nickase pairs. Nucleic Acids Res. 10.1093/nar/gku251 PMC4041425 24753413

[41] Pelascini LP, Maggio I, Liu J, Holkers M, Cathomen T, Goncalves MA (2013) Histone deacetylase inhibition rescues gene knockout levels achieved with integrase-defective lentiviral vectors encoding zinc-finger nucleases. Hum Gene Ther Methods 24: 399–411. 10.1089/hgtb.2013.107 24059449 PMC3869538

[42] Schroder AR, Shinn P, Chen H, Berry C, Ecker JR, Bushman F (2002) HIV-1 integration in the human genome favors active genes and local hotspots. Cell 110: 521–529. 12202041 10.1016/s0092-8674(02)00864-4

[43] Mitchell R, Chiang CY, Berry C, Bushman F (2003) Global analysis of cellular transcription following infection with an HIV-based vector. Mol Ther 8: 674–687. 14529841 10.1016/s1525-0016(03)00215-6

[44] Lewinski MK, Bisgrove D, Shinn P, Chen H, Hoffmann C, Hannenhalli S, et al (2005) Genome-wide analysis of chromosomal features repressing human immunodeficiency virus transcription. J Virol 79: 6610–6619. 15890899 10.1128/JVI.79.11.6610-6619.2005 PMC1112149

[45] Tebas P, Stein D, Tang WW, Frank I, Wang SQ, Lee G, et al (2014) Gene editing of CCR5 in autologous CD4 T cells of persons infected with HIV. N Engl J Med 370: 901–910. 10.1056/NEJMoa1300662 24597865 PMC4084652

[46] Mandal PK, Ferreira LM, Collins R, Meissner TB, Boutwell CL, Friesen M, et al (2014) Efficient Ablation of Genes in Human Hematopoietic Stem and Effector Cells using CRISPR/Cas9. Cell Stem Cell 15: 643–652. 10.1016/j.stem.2014.10.004 25517468 PMC4269831

